# A systematic review of the influence of rice characteristics and processing methods on postprandial glycaemic and insulinaemic responses

**DOI:** 10.1017/S0007114515001841

**Published:** 2015-08-27

**Authors:** Hanny M. Boers, Jack Seijen ten Hoorn, David J. Mela

**Affiliations:** Unilever R&D, Vlaardingen, The Netherlands

**Keywords:** Rice, Blood glucose, Insulin, Glycaemic index, Starch, Processing

## Abstract

Rice is an important staple food for more than half of the world's population. Especially in Asian countries, rice is a major contributor to dietary glycaemic load (GL). Sustained consumption of higher-GL diets has been implicated in the development of chronic diseases such as type 2 diabetes mellitus. Given that a reduction in postprandial glycaemic and insulinaemic responses is generally seen as a beneficial dietary change, it is useful to determine the variation in the range of postprandial glucose (PPG) and insulin (PPI) responses to rice and the primary intrinsic and processing factors known to affect such responses. Therefore, we identified relevant original research articles on glycaemic response to rice through a systematic search of the literature in Scopus, Medline and SciFinder databases up to July 2014. Based on a glucose reference value of 100, the observed glycaemic index values for rice varieties ranged from 48 to 93, while the insulinaemic index ranged from 39 to 95. There are three main factors that appear to explain most of the variation in glycaemic and insulinaemic responses to rice: (1) inherent starch characteristics (amylose:amylopectin ratio and rice cultivar); (2) post-harvest processing (particularly parboiling); (3) consumer processing (cooking, storage and reheating). The milling process shows a clear effect when compared at identical cooking times, with brown rice always producing a lower PPG and PPI response than white rice. However, at longer cooking times normally used for the preparation of brown rice, smaller and inconsistent differences are observed between brown and white rice.

Rice is a daily dietary staple food for more than half of the world's population, and the major single food source of carbohydrate and energy in China and many other Asian countries^(^ ^1^ ^)^. In South India, for example, nearly half of daily energy intake come from refined grains, and white polished rice constitutes >75 % of refined grain intake^(^ ^2^ ^)^. In China, brown rice is rarely consumed^(^ ^3^ ^)^. As a result, in Asian populations, white rice makes large contributions to dietary glycaemic load, an index reflecting the acute blood glucose-raising potential of foods or diets^(^ ^4^ ^)^. Higher levels of postprandial glycaemic exposure have been implicated in the development of chronic metabolic diseases, particularly type 2 diabetes mellitus and CVD^(^ ^5^ ^)^. A recent systematic review and meta-analysis has shown a clear relationship between white rice intake and the risk of type 2 diabetes mellitus, with higher levels of rice intake being more strongly associated with the risk in Asian than in Western populations^(^ ^6^ ^,^ ^7^ ^)^.

There are many varieties of rice grain in the world, which vary considerably in the postprandial blood glucose (PPG) response they produce^(^ ^8^ ^)^. The results of glycaemic index (GI) studies around the world^(^ ^9^ ^)^ report values ranging from 64 to 93. Moreover, the post-harvest treatment of rice and the method of consumer preparation can also play a significant role in this variation. Starch comprises two glucose polymers: amylose and amylopectin. Amylose is a linear and relatively short polymer of glucose units linked by α(1 → 4) bonds. Amylopectin is a branched and longer polymer where glucose units are arranged linearly through α(1 → 4), with branches emerging via α(1 → 6) bonds occurring every twenty-four to thirty glucose units^(^ ^10^ ^)^. It is well known that starches with a higher amount of amylose are more resistant to digestion^(^ ^11^ ^)^.

In addition to the variation in amylose content, cooking (and cooling) processes can influence starch digestibility via the degree of gelatinisation and retrogradation of rice starch. Gelatinisation is the collapse (disruption) of molecular order (breaking of H bonds) within the starch granule, manifested in irreversible changes in properties such as granular swelling, native crystallite melting, loss of birefringence and starch solubilisation during hydrothermal treatment^(^ ^12^ ^)^. This leads to the dissociation of crystalline regions in starch with associated hydration and swelling of starch granules, leading to higher starch availability to human digestive enzymes^(^ ^13^ ^)^. Retrogradation is the recrystallisation of amorphous phases created by gelatinisation^(^ ^14^ ^)^ and, in the case of amylose, results in the formation of type 3 resistant starch (RS3)^(^ ^15^ ^)^. RS3 is resistant to digestion, because it is heat stable and melts above 120°C^(^ ^16^ ^)^. In contrast, retrograded amylopectin is thought to melt upon reheating (cooking) due to the low melting point (46–65°C) of these crystallites, and therefore it is digestible upon cooking.

Post-harvest processing includes milling, parboiling and quick-cooking. The rice milling process starts with the husking stage to remove the husk from paddy rice, followed by the whitening–polishing stage to transform brown rice into polished white rice, and finally the grading and blending stage to obtain head rice with predefined amounts of broken rice. However, while this may affect the overall nutritional value, the effects on digestibility and PPG are less clear^(^ ^17^ ^)^. Other post-harvest treatments such as parboiling can also play a role in digestibility. Parboiling is a hydrothermal treatment that includes soaking in water, heating, drying and milling of paddy rice. During the parboiling process, the crystalline structure of the starch present in rice is transformed into an amorphous form. Pressure parboiling is accomplished by soaking paddy rice in warm water (65–68°C) for 4–5 h followed by steaming under pressure and drying^(^ ^18^ ^)^. Other post-harvest processes are used to produce quick-cooking rice. The latter is a precooked rice where the starch has been partially gelatinised by soaking in water and heating^(^ ^19^ ^)^. For consumer consumption, additional processes include cooking, storage and reheating. There are different ways of rice cooking depending on the ratios between rice and water, equipment (pressure cooking and steaming), and consumer preference (sticky rice, aromatic basmati, etc.). Cooking of polished white rice strongly affects gelatinisation. Retrogradation is affected by cooling and storage conditions (see also Fig. 3).

Given that reductions in PPG responses are generally seen as a beneficial dietary change^(^ ^5^ ^)^, it is useful to objectively establish the variation in the range of PPG responses to rice and the primary intrinsic and processing factors known to affect such responses. Therefore, we performed a systematic search of the literature characterising the range of PPG and PPI responses to different rice types, and considered this alongside available data on rice grain and processing characteristics. The main emphasis is on *in vivo* studies conducted in human subjects, supplemented in places by the *in vitro* literature related to specific mechanisms that may be relevant (e.g. influence of microstructure on rice).

## Methods

**Fig. 1:**
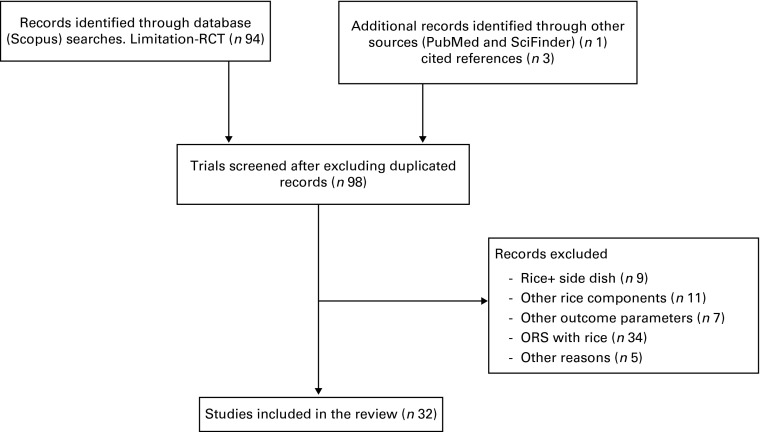
Flow chart of the systematic review article selection process. RCT, randomised controlled trial.

The literature database ‘Scopus’ was searched for the following combinations of keywords (without language or time restrictions): rice* AND glycaem* or glycem* or digestib* or glucose* or insulin* or hyperglycaem* or hyperglycem* or hypoglycaem* or hypoglycem* or normoglycaem* or normoglycem* AND combined with the title from 1980 through July 2014, resulting in ninety-four records. In addition, the PubMed and SciFinder databases were also searched using the same search terms, resulting in one additional article. A further three ‘missed’ articles were identified from the cited references in the articles identified in the formal searches, resulting in ninety-eight articles. From manual inspection of the ninety-eight abstracts, we identified twenty-eight original articles describing the results of thirty-two randomised clinical trials with rice as the test food and a measure of PPG (and in some cases also PPI) as an outcome measure (for a detailed flow chart, see Fig. 1).

## Results

### Evidence base

Studies identified in the search and their key relevant results are presented in Table 1. In addition, specific comparisons of amylose content, parboiling and milling are presented in online Supplementary Tables S2, S3 and S4, respectively. The thirty-two randomised clinical trials on PPG responses to rice included different rice types (e.g. regional varieties) and different processes (milling, (par)boiling, ‘quick-cook’ and (pressure) cooking). Outcome measures for blood glucose included GI (twenty-seven studies) and/or the incremental area under the PPG response curve (iAUC, nineteen studies), or peak glucose values (eight studies). The iAUC is the actual blood glucose response to a given serving of rice, whereas the GI and the corresponding insulinaemic index (II) use a fixed available carbohydrate load (usually 50 g) and represent responses as a comparison with a reference (assigned a value of 100). Except where noted, the GI and II studies compared rice with glucose as the reference. A subset of studies reported the II (seven studies) or insulin AUC (eight studies). Furthermore, two studies took breath hydrogen into account as an indicator of carbohydrate malabsorption^(^ ^20^ ^,^ ^21^ ^)^.

**Table 1:** Human *in vivo* studies on the postprandial glycaemic and insulinaemic effects of rice*

				Glycaemic response	
Publication+Expt	Participants	Food	Amylose (w/w%)	AUC	GI	Peak	Insulin response
Brand-Miller *et al.* (1992)^(^ ^9^ ^)^										Healthy volunteers *n* 8, age 19–36 years, BMI 18–25 kg/m^2^										Rice types grown in Australia			GI *v*. bread		II *v*. bread
min = minutes boiled					
Doongara (white), 14 min	28		64		40
Doongara (brown), 30 min	28		66		39
Pelde (brown), 30 min	20		76		55
Sunbrown (quick), 16 min	NR		80		54
Calrose (white), 14 min	20		83		67
Calrose (brown), 35 min	20		87		51
Pelde (parboiled), 14 min	20		87		57
Waxy rice, 14 min	< 2		88		89
Pelde white, 14 min	20		93		67
Ranawana *et al.* (2009)^(^ ^18^ ^)^											Healthy subjects *n* 14, age 18–65 years, BMI < 30 kg/m^2^											min = minutes boiled					
Guilin rice noodles, 8 min		76	37		
Jiangxi rice noodles, 8 min		74	40		
Easy-cook long grain rice, 15 min		76	47		
Long-grain rice (Indica type), 15 min		91	47		
White basmati rice, 10 min	20–25	94	50		
White (60 %) and brown (40 %) basmati rice, 25 min		92	59		
Basmati+wild rice, 20 min	20–25	96	63		
Brown basmati rice, 25 min	20–25	116	75		
Thai red rice, 25 min		111	76		
Easy-cook basmati rice, 15 min	20–25	111	80		
Thai glutinous rice, 10 min	< 2	144	92		
Li *et al.* (2010)^(^ ^20^ ^)^			Healthy subjects *n* 16 (*n* 9 male/*n* 7 female), age 23–26 years, BMI 18–24 kg/m^2^			RS-enriched (RS 20 %) (high amylose)					
Indica type (*Oryza sativa* L. cultivar Te-Qing)			GI	Peak	II
RS-enriched (RS 20 %, high amylose), produced with an antisense inhibition starch-branching enzyme			48	6·8	34
Wild type (RS 2%)			77	7·2	54
Casiraghi *et al.* (1993)^(^ ^21^ ^)^			Healthy subjects *n* 9, mean age 26 years, BMI 22 kg/m^2^			Italian *Fino ribe* rice, processed as:			GI *v*. bread		
Parboiled (15 min boiling time)			70		
Quick-cooking parboiled (8 min)			79		
Conventionally polished (20 min)			115		
Al-Mssallem *et al.* (2011)^(^ ^22^ ^)^		Healthy subjects *n* 13 (*n* 6 male/*n* 7 female), 25–42 years, BMI 25·6 (sem 1·0) kg/m^2^		Long-grain rice variety ‘UBR’ and traditional Saudi Arabian rice ‘HR’					
UBR	19		54		78
HR	26		59		56
Juliano & Goddard (1986) Expt 1^(^ ^23^ ^)^		*n* 16	Rice cooked: same degree of doneness		(tAUC 0–180 min)			AUC (μU/ml)
	Labelle	28	19·0†			86
	Newrex	24	19·3†			64
Juliano & Goddard (1986) Expt 2^(23)^			*n* 33	Rice cooked: same degree of doneness		(tAUC 0–180 min)			AUC (μU/ml)
	Mochi Gome	1	19·2†			113
	Labelle	24	19·3†			95
	Pecos	18	19·7†			110
Juliano *et al.* (1989)^(^ ^24^ ^)^		T2DM subjects *n* 8		Long-grain non-waxy (RD21 and RD23) and waxy rice					
Non-waxy rice	16		71		
Waxy rice	2		75		
Panlasigui *et al.* (1991) Expt 1^(25)^			Healthy subjects *n* 11 (*n* 4 male/*n* 7 female), age 23–44 years, 90–110 % ideal body weight			Long-grain, non-waxy rice: IR62, IR36 and IR42; white rice: boiled for 22 min		mmol × min/l			AUC (pmol × min/l)
IR42	26·7	55	61		9240
IR62	27	65	72		7131
IR36	26·7	81	91		9415
Panlasigue *et al.* (1991) Expt 2^(^ ^25^ ^)^				Healthy subjects *n* 11 (*n* 3 male/*n* 8 female), age 23–50 years, 90–110 % ideal body weight				Long-grain, non-waxy rice: IR62, IR36 and IR42; white rice: 50 g		mmol × min/l			
Expt 2: boiled for minimum cooking					
IR42, boiled for 14 min	26·7	26·7	81		
IR62, boiled for 20 min	27	27	75		
IR36, boiled for 19 min	26·7	26·7	78		
Panlasigui & Thompson (2006) Expt 1^(26)^		Healthy subjects *n* 10 (*n* 3 male/*n* 7 female), age 24–50 years, 90–110 % ideal body weight				mmol × min/l	GI *v*. bread		
IR42 rice, brown rice	26·7	107	83		
IR42 rice, white rice	26·7	134	94		
Panlasigui & Thompson (2006) Expt 2^(^ ^26^ ^)^		T2DM patients *n* 9 (*n* 5 male/*n* 4 female), age 45–64 years				mmol × min/l	GI *v*. bread		
IR42 rice, brown rice	26·7	406	56		
IR42 rice, white rice	26·7	626	87		
Kim *et al.* (2004)^(27)^			T2DM patients *n* 10 (*n* 4 male/*n* 6 female), mean age 57 years, BMI 24 kg/m^2^			Korean rice products:		mmol/l per 4 h			mg/dl per 4 h
Garaeduk: 16 mm stick of steamed, extruded rice flour		730			1742
Cooked rice: gelatinised grains, boiled polished rice		914			2571
Bagsulgi (rice cake): large block of steamed rice flour		1070			3266
Larsen *et al.* (2000)^(28)^				T2DM patients *n* 9, age 60 years, BMI 26·6 kg/m^2^				Indica rice variety BR16, high amylose, long grain		iAUC (mmol/l per 3 h)	GI *v*. bread		iAUC (pmol/l per 3 h)
Pressure parboiled rice	27	231	39	10·5	7590
Traditional mild parboiled rice	27	274	46	11	7719
Non-parboiled rice	27	335	55	10·9	7595
White bread		626	100	14	1652
Kataoka *et al.* (2012)^(29)^					Healthy Chinese *n* 32, age 33 years, BMI 22·9 kg/m^2^ and Healthy European subjects *n* 31, age 34 years, BMI 25·8 kg/m^2^					Rice types: jasmine rice; basmati; brown rice; Doongara; parboiled rice (Uncle Ben's)		iAUC European/Chinese (mmol × min/l)	GI European/Chinese		
Doongara	30^(^ ^9^ ^)^	109/179	55/67		
Parboiled		112/194	57/72		
Basmati	20–25^(^ ^9^ ^)^	116/184	57/67		
Brown		129/210	65/78		
Jasmine	Low^(^ ^32^ ^)^	140/225	68/80		
Trinidad *et al.* (2013)^(^ ^30^ ^)^											Healthy volunteers *n* 9–10, age 27–55 years											Cooked milled and brown rice		mmol × min/l			
Milled rice					
PSB rc10	27	188	50		
IR64	22·9	212	57		
PSB Rc18	18	221	59		
IMS2	0·6	233	63		
PSB Rc12	21	236	63		
NSIC RC160	15·3	259	70		
Sinandomeng	12·6	280	75		
Brown rice					
IR64	22	189	51		
Sinandomeng	12·1	204	55		
Zarrati *et al.* (2008)^(^ ^31^ ^)^			Healthy subjects *n* 30 (*n* 13 male/*n* 17 female), age 35 years, BMI 23 kg/m^2^			One Iranian rice type: Kazemi and imported rices				Maximum changes	II
Sorna pearl	32		52	1·2	47
Basmati	31		61	1·7	52
Kazemi	27		68	1·5	62
Larsen *et al.* (1996)^(^ ^32^ ^)^							T2DM patients *n* 12 (*n* 7 male/*n* 5 female), mean age 58 years, BMI 30 kg/m^2^							Dehulled, milled rices:		iAUC (mmol/l per 3 h)	GI *v*. bread	mmol/l	iAUC (pmol/l per 3 h)
BR2 = low amylose variety					
BR4 = low gelatinisation temperature and gel consistency *v*. BG16					
BR4-PB	27	361	47	14·5	12 964
BR16-PB	28	391	50	14·7	12 821
BR16-NP	28	411	53	14·8	11 087
BR2-PB	12	566	73	15·9	16 215
White bread		756	100	17·3	20 183
Goddard *et al.* (1984)^(^ ^33^ ^)^		*n* 33 (*n* 16 male/*n* 17 female), age 27–81 years, within 20 % desirable body weight		Long-grain rice: Labelle	23–25	19·4†		6·3	100 μU/ml
Medium-grain rice: Pecos	14–17	20·0†		6·6	105
Sweet rice: Mochi Gome	< 2	19·4†		6·8	110
Hettiarachchi *et al.* (2001)^(^ ^34^ ^)^												Healthy subjects *n* 22, age 25–50 years												Shri Lankan rice varieties (red *v*. white and parboiled *v*. raw rice)			GI *v*. bread		
Rice breeding Institute:					
Bg 350, raw, red			55		
Bw 351, parboiled, red			56		
Bw 2726-B, parboiled, red			58		
Bg 94-1, parboiled, white			62		
BW 302, raw, white			64		
Bg 300, parboiled, white			66		
Bw 400, raw, red			66		
Bg 450, raw, white			67		
Bg 94-1, raw, white			68		
Bw 2726-B, raw, red			68		
Bw 351, raw, red			73		
Srinivasa *et al.* (2013)^(^ ^35^ ^)^	Healthy volunteers *n* 83 (*n* 64 male/*n* 19 female), age 18–37 years, body weight 44–74 kg	Thermally treated Indian basmati rice		mmol × min/l		mg/l	
	182	55	76	
Henry *et al.* (2005)^(^ ^36^ ^)^			*n* 8, mean age 37 years, BMI 23 kg/m^2^			Basmati rice, Indian, boiled 8 min			69		
Basmati rice, Indian, easy-cook, boiled 9 min			67		
Basmati rice, boiled 12 min			52		
Basmati rice, organic, boiled 9 min			57		
Karupaiah *et al.* (2011)^(^ ^37^ ^)^			Healthy subjects *n* 9 (*n* 6 male/*n* 4 female), age < 30 years, BMI 23 kg/m^2^			Transgressive brown rice, cross between wild rice *O. rufipogon* Griff. and *O. sativa* L. subsp. *indica* cultivar MR219, polished version and white rice (Cap Rambutan)		mmol × min/l			II
Brown rice	13	84	51		39
Polished rice	15	130	79		63
White rice	18	141	86		68
Chiu & Stewart (2013)^(^ ^38^ ^)^			Healthy subjects *n* 21 (*n* 12 male/*n* 9 female), age 18–65 years, BMI 18·5–30·1 kg/m^2^			Refrigerated long-grain rice prepared with rice cooker (2·55 g RS/100 g as consumed) high RS					
Refrigerated short-grain rice prepared with pressure cooker (0·20 g RS/100 g) low RS					
High-RS rice		211	84		
Low-RS rice		181	78		
Wolever *et al.* (1986) Expt 1^(^ ^39^ ^)^				Diabetics *n* 18, of which NIDDM *n* 13 (*n* 6 female/*n* 7 male), age 67 years, 124 % ideal body weight and IDDM *n* 5 (*n* 4 female/*n* 1 male), age 54 years, 104 % ideal weight						NIDDM/IDDM (mmol × min/l)	NIDDM/IDDM (GI *v*. bread)	NIDDM/IDDM (mmol/l)	
White bread		951/1220	100/100	7·7/9·7	
White bread+tomato		1003/1208	107/95	8·2/9·6	
15 min regular rice	23	816/1019	86/77	6·4/7·8	
15 min parboiled rice	23	614/710	68/64	4·7/5·9	
Wolever *et al.* (1986) Expt 2^(^ ^39^ ^)^							Diabetics *n* 18, of which NIDDM *n* 13 (*n* 6 female/*n* 7 male), age 67 years; 124 % ideal body weight and IDDM *n* 5 (*n* 4 female/*n* 1 male), age 54 years, 104 % ideal body weight										GI *v*. bread		
White bread+tomato			103		
5 min regular rice			58		
15 min regular rice			83		
Instant rice			65		
5 min parboiled rice			54		
15 min parboiled rice			67		
25 min parboiled rice			66		
Jung *et al.* (2009)^(^ ^40^ ^)^			Healthy females *n* 12, mean age 22 years, BMI 21 kg/m^2^			Korean (Pungtak region) rice, processed as:					II
Uncooked rice powder			50	74	74
Freeze-dried uncooked rice powder			59	68	68
Cooked rice (boiled 15 min)			72	95	95
Parastouei *et al.* (2011)^(^ ^51^ ^)^		Healthy young adults *n* 10, mean age 20 years, BMI 20 kg/m^2^		‘Iranian’ white rice (no further details on type):					
Fluffy (soaked 35 min → boiled 10 min → drained and simmered 20–30 min)			55		
Steamed (boiled 5–8 min → simmered 30 min)			66		
Truong *et al.* (2014)^(^ ^57^ ^)^				Healthy volunteers *n* 12 (*n* 9 female/*n* 3 male), age 18–65 years, BMI 23 kg/m^2^				Four brands of Jasmine rice:					
Della (USA)	Low		96		
Jazzmen (USA)	Low		106		
Reindeer (Thailand)	Low		115		
Mahatma (Thailand)	Low		116		
Gatti *et al.* (1987)^(^ ^59^ ^)^		Healthy subjects *n* 14 (*n* 9 male/*n* 5 female), age 21–32 years, body weight 88–115 kg		Rice was cooked in two different ways:		60 min			AUC (U/ml)
Boiled in salt water		61			2536
Baked for 10 min at 160°C after boiling		43			2676
Matsuo *et al.* (1999) Expt 1^(^ ^60^ ^)^	Healthy adults *n* 8 (*n* 3 male/*n* 5 female), mean age 25 years, BMI 20 kg/m^2^	Short-grain Koshihikari rice			48		II = 65
3 h GI and II *v*. glucose reference					
Shobana *et al.* (2012)^(^ ^61^ ^)^			Healthy volunteers *n* 23, age 18–45 years, BMI < 23·0 kg/m^2^			Indian rice varieties (Sona Masuri, Ponni and Surti Kolam)		mmol × min/l			
Ponni		175	70		
Sona Masuri		172	72		
Surti Kolam		185	77		

GI, glycaemic index; II, insulinaemic index; NR, not reported; RS, resistant starch; UBR, Uncle Ben's rice; HR, Hassawi rice; tAUC, total AUC; T2DM, type 2 diabetes mellitus; iAUC, incremental AUC; PB, parboiled; NP, not parboiled; Bg, Bathalagaoda; Bw, Bombuwala; NIDDM, non-insulin-dependent diabetes mellitus; IDDM, insulin-dependent diabetes mellitus.

* For the GI and II values, 50 g of available carbohydrates were used, with glucose as the reference (except where noted) being assigned the value of 100.

† The AUC was not calculated by the trapezoidal method but by the following formula: (time 1)/4+(time 2)/2+¾ time 3+time 4+time 5.

### Characterisation of rice and processing

**Fig. 2:**
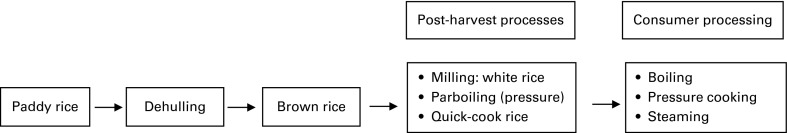
Rice processing steps.

In most studies, rice was well characterised with respect to the percentage of amylose (nine studies), dietary fibre (four studies), RS (two studies) and available starch (sixteen studies). In some studies, gelatinisation or amylograph measurements of milled rice flour were taken into account^(^ ^22^ ^–^ ^26^ ^)^, while in others, *in vitro* glucose release assays were included^(^ ^21^ ^,^ ^24^ ^,^ ^27^ ^)^. A few studies reported grain size, rheology or retrogradation determined by differential scanning calorimetry (a thermo-analytical technique to identify phase transition)^(^ ^28^ ^)^. The processes explored in the studies involved post-harvest treatments such as parboiling and milling (Fig. 2) .

Variation observed in the glycaemic index and insulinaemic index and its causes

The observed GI values ranged from 48 to 93, while the II values (0–120 min) ranged from 39 to 95 (Table 1).

In the studies that specifically tested or varied the amylose content and its quantitative relationship with glycaemic and insulinaemic responses^(^ ^9^ ^,^ ^18^ ^,^ ^20^ ^,^ ^22^ ^,^ ^23^ ^,^ ^29^ ^–^ ^33^ ^)^, the latter measures were significantly inversely associated with the amylose content^(^ ^9^ ^,^ ^18^ ^,^ ^20^ ^,^ ^29^ ^–^ ^32^ ^)^ (see also online Supplementary Table S2). However, some studies did not find this inverse relationship for all glycaemic parameters^(^ ^22^ ^,^ ^23^ ^,^ ^33^ ^)^. Large differences in amylose content (2 % *v*. approximately 30 % amylose) were often associated with relatively large glycaemic and insulinaemic effects (approximately 300 % decrease in PPG; approximately 55 % decrease in PPI)^(^ ^9^ ^,^ ^18^ ^,^ ^29^ ^)^. However, there were also studies in which this effect was inconsistent^(^ ^30^ ^)^ or not observed^(^ 23 (Expt 2) ^,^ ^33^ ^)^.

Rice that received post-harvest treatments such as parboiling^(^ ^21^ ^,^ ^29^ ^,^ ^34^ ^)^ and quick-cooking^(^ ^18^ ^,^ ^21^ ^)^ generally gave a lower GI compared with white rice not subjected to these post-harvest treatments (see also online Supplementary Table S3). Larsen *et al.* ^(^ ^28^ ^)^ reported that an increased severity of parboiling conditions leads to significant decreases in PPG responses due to the formation of RS. In that study, mild traditional parboiling had no effect on the GI, whereas severely pressure parboiling reduced the GI by almost 30 % compared with non-parboiled rice. However, one study did not show an effect of parboiling^(^ ^32^ ^)^, and the reported GI of a thermally treated Indian basmati rice variety (thermal treatment not specified) was 55^(^ ^35^ ^)^, which was in the range between 52 and 59 reported for non-thermally treated Indian basmati rice by Henry *et al.* ^(^ ^36^ ^)^. The influence of another post-harvest treatment, milling, by which brown rice is transformed into white rice, was considered in several studies^(^ ^9^ ^,^ ^18^ ^,^ ^26^ ^,^ ^30^ ^,^ ^37^ ^)^ (see online supplementary Table S4). In those studies where cooking times were identical^(^ ^26^ ^,^ ^30^ ^,^ ^37^ ^)^, brown rice always produced lower PPG and PPI responses. However, when realistic (longer) cooking times were applied to brown rice^(^ ^9^ ^,^ ^18^ ^)^, the difference between brown and white rice was smaller and inconsistent.

Consumer processing can also make a large contribution to the formation of RS in rice. Chiu & Stewart^(^ ^38^ ^)^ quantified RS content in four white rice varieties (jasmine, long grain, medium grain and short grain) cooked in three different ways (oven-baked, conventional rice cooker and pressure cooker), and analysed the RS content immediately after preparation or after 3 d of refrigeration at 4°C. Refrigerated long-grain rice cooked in a conventional rice cooker had the highest RS content, while the refrigerated short-grain rice cooked in a pressure cooker had the lowest RS content. However, in this case, the GI values did not differ significantly between the higher-RS and lower-RS rice varieties. Consumer processing can also have a large effect on gelatinisation. Wolever *et al.* ^(^ ^39^ ^)^ showed that the GI generally increased with cooking time for rice, while Jung *et al.* ^(^ ^40^ ^)^ showed a marked increase in gelatinisation upon cooking rice and a somewhat higher GI and II.

## Discussion

**Fig. 3:**
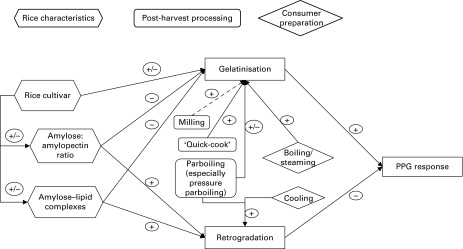
Relationship between rice characteristics, processing factors, physico-chemical processes and glycaemic response (+ indicates increased effect; −  indicates decreased effect). This is a general figure, depending on specific processes, e.g. conditions of parboiling; the effects may differ. PPG, postprandial glucose response.

The literature reveals considerable variation in the glycaemic or insulin response to rice. This is largely attributable to (1) starch characteristics, (2) post-harvest processing (particularly parboiling and to a much lesser extent dehulling and milling) and (3) consumer processing (cooking, storage and reheating). The relationships among rice characteristics and processing factors, and their physico-chemical effects and impact on glycaemic responses are qualitatively shown in Fig. 3.

### Influence of the composition and processing of rice

The most consistently important source of variation in PPG responses to rice is amylose content. The amylose content of rice varies between 0 % (waxy rice) and 30 % (Doongara)^(^ ^9^ ^)^, with basmati having an intermediate value (20–25 % amylose^(^ ^41^ ^)^). One of the reasons for the lower PPG responses to high amylose varieties is incomplete gelatinisation of amylose under normal cooking conditions, while amylopectin is fully gelatinised under these conditions^(^ ^42^ ^)^. Gelatinisation temperature is known to be positively correlated with amylose content^(^ ^43^ ^)^, implying that rice with a higher amylose content requires a higher gelatinisation temperature due to restrained swelling by amylose, resulting in a longer required cooking time^(^ ^44^ ^)^. The formation of complexes between amylose and lipids upon heating further contributes to reduced access to starch by gut enzymes^(^ ^33^ ^)^. These complexes with lipids are only found in association with amylose; therefore, rice with the highest amylose content would have more lipid–amylose complexes^(^ ^33^ ^)^. In addition, a higher amylose content (after cooking and cooling) leads to a greater degree of retrogradation^(^ ^18^ ^)^. A recent study found the major gene associated with the variation in the GI was the waxy gene^(^ ^44^ ^)^, which codes for different structures of amylose within the grain and leads to different retrogradation rates^(^ ^45^ ^)^.

The *in vitro* literature showed that the rice cultivar, clustered as Indica, Japonica and Hybrid rice type, plays a pivotal role in the rate and degree of starch digestion: low-amylose Indica showed a faster and higher degree of digestion than low-amylose Japonica, while a high-amylose Japonica was faster and more completely digested (reflected by a higher content of rapidly digestible starch and a lower content of slowly digestible starch and RS) than high-amylose Indica^(^ ^11^ ^)^. In addition, Benmoussa *et al.* ^(^ ^46^ ^)^ showed that amylopectin fine structure in rice cultivars affects starch digestion properties *in vitro*: cultivars with the highest amount of slowly digestible starch contained mainly long-chain amylopectin.

Post-harvest treatments such as parboiling^(^ ^21^ ^,^ ^29^ ^,^ ^34^ ^)^ and quick-cooking^(^ ^18^ ^,^ ^21^ ^)^ also have a large influence on the GI (see online Supplementary Table S3). Gelatinisation and re-crystallisation are the major changes that occur in rice starch during parboiling^(^ ^47^ ^)^. The parboiling process increases the gelatinisation temperature of rice that is proportional to the severity of the heat treatment^(^ ^48^ ^)^. This is probably the reason why pressure parboiling lowers the GI to such a large extent, especially of high-amylose starches^(^ ^49^ ^)^. The pressure parboiling process increases gelatinisation temperature due to the formation of retrograded amylose and amylopectin. Wet heating and subsequent drying during these processes result in the gelatinisation of starch, followed by retrogradation of amylose and amylopectin^(^ ^18^ ^)^ leading to higher levels of RS. It is possible that amylopectin crystallites (part of RS) retain some of the associating forces during reheating, and are partly responsible for the low glucose response observed during pressure parboiling. The amylose–lipid complexes have a melting temperature above 100°C and are not melted during the cooking process, resulting in higher levels of RS^(^ ^28^ ^)^.

Another way of achieving a high RS content is to apply multiple heating/cooling cycles^(^ ^50^ ^)^. After three heating/cooling cycles, the RS content of legumes, cereals and tubers increased from 4·18, 1·86 and 1·51 % to 8·16, 3·25 and 2·51 %, respectively, on a DM basis. However, a ten times greater RS content in rice varieties had no effect on the GI^(^ ^38^ ^)^. It is possible that the tested range of difference in RS content in that study was not sufficient to observe a change in the GI^(^ ^38^ ^)^, which is confirmed by the fact that only large differences in amylose content (leading to high RS content after cooking and cooling) lead to relatively large effects on the GI^(^ ^9^ ^)^.

Another final process shown to have a major influence on the PPG response is the gelatinisation process during cooking, which needs moisture and a high temperature (above gelatinisation temperature) for a particular period of time. Using different rice types with the same high amylose content, Panlasigui *et al.* ^(^ ^25^ ^)^ reported that PPG responses differed between rice types when a fixed cooking time was used; however, these differences disappeared when the minimum cooking time for each particular rice type was used. This is likely attributed to other physico-chemical properties of rice types. Physico-chemical parameters that predict lower blood glucose responses are high gelatinisation temperature, high minimum cooking time, lower viscosity measured by amylograph consistency (amylograph is an instrument for measuring gelatinisation temperature and viscosity of flour and starch pastes), and low volume expansion upon cooking, all parameters relating to lower gelatinisation^(^ ^25^ ^)^. Steaming also gave a larger PPG response than boiling and simmering^(^ ^51^ ^)^, which may reflect greater gelatinisation by steaming.

A factor that has a relatively less impact on PPG responses is physical size and form of the whole kernel rice, probably due to the fact that size is minimised by chewing^(^ ^52^ ^)^. Particle size only plays a major role when the rice is milled to rice flour, resulting in the higher surface area:starch ratio that leads to an increased rate of digestion^(^ ^53^ ^)^. In addition, the effect of brown rice *v*. white rice on glycaemic and insulinaemic responses shows a clear difference^(^ ^26^ ^,^ ^30^ ^,^ ^37^ ^)^ when compared at identical cooking times: for instance, brown rice always gives a lower PPG and PPI response (see online Supplementary Table S4). However, in reality, consumers cook brown rice longer than white rice, resulting in a mixed outcome: in some cases, white rice was found to have a higher glycaemic response^(^ ^9^ ^)^ (for Pelde), or a neutral effect^(^ ^9^ ^)^ (for Doongara and Calrose) or even a lower response than brown rice^(^ ^18^ ^)^. In most of these studies^(^ ^9^ ^,^ ^18^ ^,^ ^30^ ^)^ commercially available white rice was taken at random and not milled from the same batch of brown rice. Therefore, the variety and physico-chemical properties of rice samples may have differed^(^ ^53^ ^)^. Only two studies^(^ ^26^ ^,^ ^37^ ^)^ used white and brown rice from the same batch. However, a recent longer-term study showed that the iAUC over 5 d consumption was 19·8 % lower for a group eating brown *v*. white rice, as measured with a continuous glucose monitoring device^(^ ^54^ ^)^. However, it is not clear whether brown rice and white rice were of the same rice variety. Therefore, the results cannot clearly be attributed to the milling process alone. It is possible that the dietary fibre-rich bran fraction in brown rice can continue to serve as a barrier to digestive enzymes^(^ ^53^ ^)^, but several other modes of action are also possible. The magnitude of the effect of milling and polishing could also be somewhat dependent on the rice strain and cooking conditions^(^ ^18^ ^)^. White rice has a shorter minimum cooking time and higher volume expansion than brown rice, indicating that white rice is more easily hydrated and gelatinised compared with brown rice, and therefore more readily digested resulting in a higher PPG response^(^ ^53^ ^)^ when cooked under the same conditions.

In addition to the rice source and processing, there is an inter-individual variation observed in PPG (iAUC and peak blood glucose) responses to carbohydrate-rich foods. This was reported to account for at least 20 % of the total variation in PPG responses^(^ ^55^ ^)^. One of the factors that could be responsible for the inter-individual variation in PPG responses to rice could be ethnicity. The PPG (+iAUC) response was 60 % greater for five rice varieties and 39 % greater for glucose among the Chinese population compared with Europeans^(^ ^29^ ^)^ (Table 1). The most likely explanation for these ethnic differences is that the Chinese population are more likely to become insulin resistant than Europeans of the same or higher relative body weight and waist circumference^(^ ^56^ ^)^. Truong *et al.* ^(^ ^57^ ^)^ also observed that Asian Americans on average exhibited higher levels of blood glucose than Caucasians after consumption of a control food with 50 g carbohydrates. Therefore, when comparing the results across studies, ethnicity of the subjects should be taken into account: i.e. Asian people typically have a higher PPG response than Caucasians, which may also increase the apparent magnitude of differences between rice types and characteristics.

A final factor contributing towards the inter-individual variation in PPG responses is the degree of habitual mastication^(^ ^52^ ^)^. The latter may be a considerable contributor, especially to foods consisting of intact grains (such as rice) that rely on mechanical breakdown for carbohydrate release. Indeed, a recent study^(^ ^58^ ^)^ showed that rice chewed fifteen times produced a PPG, peak PPG and GI response significantly lower than that when chewed thirty times.

### Conclusions

While rice as a total category may be a major global contributor to dietary glycaemic load, there is a wide variation in glycaemic and insulinaemic responses to rice as consumed. This can be largely attributed to the inherent starch characteristics of specific cultivars; however, within a given rice type, the mode of post-harvesting processing and ‘at-home’ preparation can also have a large influence. A reduced glycaemic impact is mediated mainly by the relative content of amylose (*v*. amylopectin), reduction in gelatinisation, or the facilitation of retrogradation. Perhaps, surprisingly, milling and polishing (thus white *v*. brown rice) has been found to have inconsistent impacts on acute glycaemic responses when compared at realistic cooking times that are longer for brown rice. The glycaemic response to rice can be further influenced by individual characteristics of the consumer, such as chewing habit and ethnicity. In order to interpret and compare the reported PPG responses between different studies in rice, the rice cultivar, amylose:amylopectin ratio, post-harvest processing parameters and cooking conditions should be considered. In addition, a lower PPG response to rice can be achieved by choosing right conditions, for example high amylose content, minimised cooking times (or pressure parboiled) and cooled before consumption. The opposite effect (a higher PPG response) can be achieved by selecting for low-amylose (waxy) white rice, with a long cooking time, and consuming directly after cooking.

## Supplementary material

For supplementary material accompanying this paper visit http://dx.doi.org/10.1017/S0007114515001841. click here to view supplementary material
