# Supplementary material for: A systematic review of the influence of rice characteristics and processing methods on postprandial glycaemic and insulinaemic responses
Source: Br J Nutr. 2015 Aug 27;114(7):1035–45. doi: 10.1017/S0007114515001841 (PMC4579564; doi:10.1017/S0007114515001841)
Supplement: Supplementary file 1 [file S0007114515001841sup001.zip › S0007114515001841sup/S0007114515001841sup001.docx]

Table 2: Glycaemic and insulin response data classified by Inherent characteristics (e.g. amylose content + grain type + variety/name), post-harvest and consumer processing.

| **Inherent characteristics** | | | **Process**  **Post-harvest** | **Process**  **consumer** | | **Glycaemic response** | | | **Insulin** | **Publication** |
| --- | --- | --- | --- | --- | --- | --- | --- | --- | --- | --- |
| Amylose | Grain type | Variety |  | Cooker | Boiling  time [m] | AUC | GI | Peak  [mmol/l] |  |  |
| **Waxy (<2%)** | | | | | | | | | | |
|  |  | Moche Gome |  |  |  | 19^[[1]](#footnote-1)^ |  | 6.8 | 110 | Goddard-1984^(33)^ |
|  |  | Moche Gome |  | Degree of doneness | | 19^[[2]](#footnote-2)^ |  |  | 113^2^ | Juliano-1986-2^(23)^ |
|  |  | RD23 |  |  |  |  | 75 |  |  | Juliano-1989^(24)^ |
|  |  | Thai glutinous | white |  | 10 | 144 | 92 |  |  | Ranawana-2009^(18)^ |
| 0.6 |  | IMS2 | milled | cooked |  | 233^[[3]](#footnote-3)^ | 63 |  |  | Trinidad-2013^(30)^ |
| **Low (12 – 20%)** | | | | | | | | | | |
| 12 |  | BR2 | parboiled |  |  | 566^[[4]](#footnote-4)^ | 100 | 15.9 | 16215^4^ | Larsen-1996^(32)^ |
| 12.1 |  | Sinandomeng | brown | cooked |  | 204^3^ | 55 |  |  | Trinidad-2013^(30)^ |
| 12.6 |  | Sinandomeng | milled | cooked |  | 280^3^ | 75 |  |  | Trinidad-2013^(30)^ |
| 13 |  | MR219 | brown |  |  | 84 | 51 |  | 39 | Karupaiah-2011^(37)^ |
| 15 |  | MR219 | polished |  |  | 130 | 79 |  | 63 | Karupaiah-2011^(37)^ |
| 15.3 |  | NSIC RC160 | milled | cooked |  | 259^3^ | 70 |  |  | Trinidad-2013^(30)^ |
| 16 | Long | RD21 |  |  |  |  | 71 |  |  | Juliano-1989^(24)^ |
| 14-17 | medium | Pecos |  |  |  | 20^1^ |  | 6.6 | 105^1^ | Goddard-1984^(33)^ |
| 18 |  | Pecos |  | Degree of doneness | | 20^2^ |  |  | 110^2^ | Juliano-1986-2^(23)^ |
| 18 |  | Cap Rambutan | white |  |  | 141 | 86 |  | 68 | Karupaiah-2011^(37)^ |
| 18 |  | PSB Rc18 | milled | cooked |  | 221^3^ | 59 |  |  | Trinidad-2013^(30)^ |
| 19 | long | Uncle Ben’s | parboiled | Rice:water = 1:2 | 17 |  | 54 |  | 78 | Al-Mssallem-2011^(22)^ |
| NR |  | Uncle Ben’s | parboiled | Rice cooker |  | 194 | 72 |  |  | Kataoka-2012-Chinese^(29)^ |
| NR |  | Uncle Ben’s | parboiled | Rice cooker |  | 112 | 57 |  |  | Kataoka-2012-European^(29)^ |
| NR | Aromatic | Thai Jasmine | White | Rice cooker |  | 225 | 80 |  |  | Kataoka-2012-Chinese^(29)^ |
| NR | Aromatic | Thai Jasmine | White | Rice cooker |  | 140 | 68 |  |  | Kataoka-2012-European^(29)^ |
| 20 |  | Pelde | brown |  | 30 |  | 76 |  | 55 | Brand-Miller-1992^(9)^ |
| 20 |  | Pelde | parboiled |  | 14 |  | 87 |  | 57 | Brand-Miller-1992^(9)^ |
| 20 |  | Pelde | white |  | 14 |  | 93 |  | 67 | Brand-Miller-1992^(9)^ |
| 20 |  | Calrose | Brown |  | 35 |  | 87 |  | 51 | Brand-Miller-1992^(9)^ |
| 20 |  | Calrose | white |  | 14 |  | 83 |  | 67 | Brand-Miller-1992^(9)^ |
| low | Jasmine | Della | white | cooked |  |  | 96 |  |  | Truong-2014^(57)^ |
| low | Jasmine | Jazzmen | white | cooked |  |  | 106 |  |  | Truong-2014^(57)^ |
| low | Jasmine | Reindeer | white | cooked |  |  | 115 |  |  | Truong-2014^(57)^ |
| low | Jasmine | Mahatma | white | cooked |  |  | 116 |  |  | Truong-2014^(57)^ |
| **Intermediate (20 – 25%)** | | | | | | | | | | |
| 21.0 |  | PSB Rc12 | milled | cooked |  | 236^3^ | 63 |  |  | Trinidad-2013^(30)^ |
| 22.0 |  | IR64 | brown | cooked |  | 189^3^ | 51 |  |  | Trinidad-2013^(30)^ |
| 22.9 |  | IR64 | milled | cooked |  | 212^3^ | 57 |  |  | Trinidad-2013^(30)^ |
| 23 |  | “regular” | white | boiling | 15 | 816^[[5]](#footnote-5)^ | 86 | 6.4 |  | Wolever-1986-1-NIDDM^(39)^ |
| 23 |  | “regular” | white | boiling | 15 | 1019^5^ | 77 | 7.8 |  | Wolever-1986-1-IDDM^(39)^ |
| 23 |  | “regular” | parboiled | boiling | 15 | 614^5^ | 68 | 4.7 |  | Wolever-1986-1-NIDDM^(39)^ |
| 23 |  | “regular” | parboiled | boiling | 15 | 710^5^ | 64 | 5.9 |  | Wolever-1986-1-IDDM^(39)^ |
| 23 |  | “regular” | white | boiling | 5 |  | 58 |  |  | Wolever-1986-2^(39)^ |
| 23 |  | “regular” | white | boiling | 15 |  | 83 |  |  | Wolever-1986-2^(39)^ |
| 23 |  | “regular” | parboiled | boiling | 5 |  | 54 |  |  | Wolever-1986-2^(39)^ |
| 23 |  | “regular” | parboiled | boiling | 15 |  | 67 |  |  | Wolever-1986-2^(39)^ |
| 23 |  | “regular” | parboiled | boiling | 25 |  | 66 |  |  | Wolever-1986-2^(39)^ |
| 23-25 | long | Labelle |  |  |  | 19^1^ |  | 6.3 | 100^1^ | Goddard-1984^(33)^ |
| 24 | long | Labelle |  |  |  | 19^2^ |  |  | 86^2^ | Juliano-1986-1^(23)^ |
| 24 | long | Labelle |  |  |  | 19^2^ |  |  | 95^2^ | Juliano-1986-2^(23)^ |
|  | Indian | Basmati | White | boiling | 8 |  | 69 |  |  | Henry-2005^(36)^ |
|  | Indian | Basmati | White | Boiling | 12 |  | 52 |  |  | Henry-2005^(36)^ |
|  |  | Basmati | Easy-cook | boiling | 9 |  | 67 |  |  | Henry-2005^(36)^ |
|  | Organic | Basmati | White (?) | boiling | 9 |  | 57 |  |  | Henry-2005^(36)^ |
|  |  | Basmati | White | Rice cooker |  | 184 | 67 |  |  | Kataoka-2012-Chinese^(29)^ |
|  |  | Basmati | White | Rice cooker |  | 116 | 57 |  |  | Kataoka-2012-European^(29)^ |
|  |  | Basmati | Thermal-treated |  |  | 182^[[6]](#footnote-6)^ | 55 | 7.6^6^ |  | Srinivasa-2013^(35)^ |
|  |  | Ponni | White | Rice:water = 1:3.5 | 35 | 175 | 70 |  |  | Shobana-2012^(61)^ |
|  | medium | Sona Masuri | White | Rice:water = 1:3.5 | 35 | 172 | 72 |  |  | Shobana-2012^(61)^ |
|  |  | Surti Kolam | White | Rice:water = 1:3.5 | 35 | 185 | 77 |  |  | Shobana-2012^(61)^ |
| **High (25 – 33%)** | | | | | | | | | | |
| 26 |  | Hassawi | Brown | Rice:water=1:2 | 45 |  | 59 |  | 56 | Al-Mssallem-2011^(22)^ |
| 26.7 | Long | IR36 | White | boiling | 22 | 81^3^ | 91 |  | 9415^3^ | Panlasigui-1991-1^(25)^ |
| 26.7 | Long | IR36 | White | boiling | 19 |  | 78 |  |  | Panlasigui-1991-2^(25)^ |
| 26.7 | Long | IR42 | White | boiling | 22 | 55^3^ | 61 |  | 9240^3^ | Panlasigui-1991-1^(25)^ |
| 26.7 | Long | IR42 | White | boiling | 14 |  | 91 |  |  | Panlasigui-1991-2^(25)^ |
| 26.7 | Long | IR42 | Brown |  |  | 107^3^ | 83 |  |  | Panlasigui-2006-healthy^(26)^ |
| 26.7 | Long | IR42 | White |  |  | 134^3^ | 94 |  |  | Panlasigui-2006-healthy^(26)^ |
| 26.7 | Long | IR42 | Brown |  |  | 406 | 56 |  |  | Panlasigui-2006-T2DM^(26)^ |
| 26.7 | Long | IR42 | white |  |  | 626 | 91 |  |  | Panlasigui-2006-T2DM^(26)^ |
| 27.0 | long | IR62 | white | boiling | 22 | 65^3^ | 72 |  | 7131^3^ | Panlasigui-1991-1^(25)^ |
| 27.0 | long | IR62 | white | boiling | 20 |  | 75 |  |  | Panlasigui-1991-2^(25)^ |
| 27.0 |  | PSB rc10 | milled | cooked |  | 188^3^ | 50 |  |  | Trinidad-2013^(30)^ |
| 27 |  | BR4 | parboiled |  |  | 361^4^ | 47 | 14.5 | 12964^4^ | Larsen-1996^(32)^ |
| 28 |  | BR16 | Parboiled |  |  | 391^4^ | 50 | 14.7 | 12821^4^ | Larsen-1996^(32)^ |
| 28 |  | BR16 | polished |  |  | 411^4^ | 53 | 14.8 | 11087^4^ | Larsen-1996^(32)^ |
| 27 | Indica-long | BR16 | Pressure  parboiled |  |  | 231^4^ | 39 | 10.5 | 7590^4^ | Larsen-2000^(28)^ |
| 27 | Indica-long | BR16 | Mild parboiled |  |  | 274^4^ | 46 | 11.0 | 7719^4^ | Larsen-2000^(28)^ |
| 27 | Indica-long | BR16 | Polished |  |  | 335^4^ | 55 | 10.9 | 7595^4^ | Larsen-2000^(28)^ |
| 28 |  | Newrex |  | Degree of doneness | | 19^2^ |  |  | 64^2^ | Juliano-1986-1^(23)^ |
| 28 |  | Doongara | Brown | boiling | 30 |  | 66 |  | 39 | Brand-Miller-1992^(9)^ |
| 28 |  | Doongara | white | boiling | 14 |  | 64 |  | 40 | Brand-Miller-1992^(9)^ |
| 28 |  | Doongara | white |  |  | 179 | 67 |  |  | Kataoka-2012-Chinese^(29)^ |
| 28 |  | Doongara | white |  |  | 109 | 55 |  |  | Kataoka-2012-European^(29)^ |
| 27 |  | Kazemi |  |  |  |  | 68 | 27mg/dl | 62 | Zarrati-2008^(31)^ |
| 31 |  | Basmati |  |  |  |  | 61 | 32mg/dl | 52 | Zarrati-2008^(31)^ |
| 32 |  | Sorna pearl |  |  |  |  | 52 | 22mg/dl | 47 | Zarrati-2008^(31)^ |
|  |  |  |  |  |  |  |  |  |  |  |

1. AUC expressed as mmol/l, and insulin response expressed as µU/ml [↑](#footnote-ref-1)
2. AUC expressed as mmol/l; tAUC 0-180 min., and insulin response expressed as µU/ml [↑](#footnote-ref-2)
3. AUC expressed as mmol/l, and insulin response expressed as pmol/l. [↑](#footnote-ref-3)
4. AUC expressed as iAUC mmol/l/3hr, and insulin response expressed as pmol/l/3hr [↑](#footnote-ref-4)
5. AUC expressed as mmol/l [↑](#footnote-ref-5)
6. AUC expressed as mmol.min/l., and Peak expressed as mmol/l [↑](#footnote-ref-6)
