# Supplementary material for: A systematic review of the influence of rice characteristics and processing methods on postprandial glycaemic and insulinaemic responses
Source: Br J Nutr. 2015 Aug 27;114(7):1035–45. doi: 10.1017/S0007114515001841 (PMC4579564; doi:10.1017/S0007114515001841)
Supplement: Supplementary file 1 [file S0007114515001841sup001.zip › S0007114515001841sup/S0007114515001841sup002.docx]

Table 3: Glycaemic and insulin response classified by (pressure) parboiled versus non-parboiled rice and quick cook rice

| **(Severely) parboiled versus non parboiled rice**  Min = minutes boiled | **Variety** | **Glycaemic response** | | | **Insulin** | **Publication** |
| --- | --- | --- | --- | --- | --- | --- |
|  |  | **AUC** | GI  *vs bread | **Peak** |  |  |
| Parboiled, 14 min | Pelde (white) |  | 87* |  | 57 | Brand-Miller-1992^(9)^ |
| Non-parboiled, 14 min | Pelde (white) |  | 93* |  | 67 | Brand-Miller-1992^(9)^ |
| Parboiled, 15 min | Italian Fino ribe |  | 70* |  |  | Casiraghi-1993^(21)^ |
| Quick-cooking parboiled, 8 min | Italian Fino ribe |  | 79* |  |  | Casiraghi-1993^(21)^ |
| Non-parboiled, 20 min | Italian Fino ribe |  | 115* |  |  | Casiraghi-1993^(21)^ |
| Parboiled | BW 351, red |  | 56* |  |  | Hettiarachchi-2001^(34)^ |
| Non-parboiled | BW 351, raw, red |  | 73* |  |  | Hettiarachchi-2001^(34)^ |
| Parboiled | BW 2726-B, red |  | 58* |  |  | Hettiarachchi-2001^(34)^ |
| Non-parboiled | BW 2726-B, raw, red |  | 68* |  |  | Hettiarachchi-2001^(34)^ |
| Parboiled | Bg 94-1, white |  | 62* |  |  | Hettiarachchi-2001^(34)^ |
| Non-parboiled | Bg-94-1, raw, white |  | 68* |  |  | Hettiarachchi-2001^(34)^ |
| Parboiled | Parboiled (Uncle Ben’s, Masterfoods) | 112 | 57 |  |  | Kataoka-2012-European^(29)^ |
| Parboiled | Parboiled (Uncle Ben’s, Masterfoods) | 194 | 72 |  |  | Kataoka-2012-Chinese^(29)^ |
| Parboiled | Indica rice BR16 | 391 | 50* | 14.7 | 12821 | Larsen-1996^(32)^ |
| Non-parboiled | Indica rice BR16 | 411 | 53* | 14.8 | 11087 | Larsen-1996^(32)^ |
| Pressure parboiled | Indica rice BR16 | 231 | 39* | 10.5 | 7590 | Larsen-2000^(28)^ |
| Traditional mild parboiled | Indica rice BR16 | 274 | 46* | 11.0 | 7719 | Larsen-2000^(28)^ |
| Non-parboiled | Indica rice BR16 | 335 | 55* | 10.9 | 7595 | Larsen-2000^(28)^ |
| Easy-cook | Basmati rice, 15 min | 111 | 80 |  |  | Ranawana-2009^(18)^ |
| white rice (non-easy cook) | Basmati rice, 10 min | 94 | 50 |  |  | Ranawana-2009^(18)^ |
| Easy-cook | Long grain, Indica, 15 min | 76 | 47 |  |  | Ranawana-2009^(18)^ |
| Non-easy cook | Long grain, Indica, 15 min | 91 | 47 |  |  | Ranawana-2009^(18)^ |
| Parboiled | Regular long-grain | 614 | 68* | 4.7 |  | Wolever-1986-NIDDM-1^(39)^ |
| Non-parboiled | Regular long-grain | 816 | 86* | 6.4 |  | Wolever-1986-NIDDM-1^(39)^ |
| Parboiled | Regular long-grain | 710 | 64* | 5.9 |  | Wolever-1986-IDDM-1^(39)^ |
| Non-parboiled | Regular long-grain | 1019 | 77* | 7.8 |  | Wolever-1986-IDDM-1^(39)^ |
| Non-parboiled | Regular, 5 min |  | 58* |  |  | Wolever-1986-NIDDM-2^(39)^ |
| Parboiled | Regular, 5 min |  | 54* |  |  | Wolever-1986-NIDDM-2^(39)^ |
| Non-parboiled | Regular, 15 min. |  | 83* |  |  | Wolever-1986-NIDDM-2^(39)^ |
| Parboiled | Regular, 15 min |  | 67* |  |  | Wolever-1986-NIDDM-2^(39)^ |
| Parboiled | Regular, 25 min |  | 66* |  |  | Wolever-1986-NIDDM-2^(39)^ |
