# Supplementary material for: A systematic review of the influence of rice characteristics and processing methods on postprandial glycaemic and insulinaemic responses
Source: Br J Nutr. 2015 Aug 27;114(7):1035–45. doi: 10.1017/S0007114515001841 (PMC4579564; doi:10.1017/S0007114515001841)
Supplement: Supplementary file 1 [file S0007114515001841sup001.zip › S0007114515001841sup/S0007114515001841sup003.docx]

Table 4: glycaemic and insulin response classified by state of milling

| **Milling state (brown versus white)** min = min boiled | **Variety** | **Glycaemic response** | | | **Insulin**  ** II vs bread | **Publication** |
| --- | --- | --- | --- | --- | --- | --- |
|  |  | **AUC** | GI  *vs bread | **Peak** |  |  |
| White, 14 min. | Doongara |  | 64* |  | 40** | Brand-Miller-1992^(9)^ |
| Brown, 30 min | Doongara |  | 66* |  | 39** | Brand-Miller-1992^(9)^ |
| White, 14 min | Pelde |  | 93* |  | 93** | Brand-Miller-1992^(9)^ |
| Brown, 30 min | Pelde |  | 76* |  | 76** | Brand-Miller-1992^(9)^ |
| White, 14 min | Calrose |  | 83* |  | 67** | Brand-Miller-1992^(9)^ |
| Brown, 35 min | Calrose |  | 87* |  | 51** | Brand-Miller-1992^(9)^ |
| White | Transgressive | 130 | 79 |  | 63 | Karupaiah-2011^(37)^ |
| Brown | Transgressive | 84 | 51 |  | 39 | Karupaiah-2011^(37)^ |
| White | IR42 | 134 | 94* |  |  | Panlasigui and Thompson-2006-1-healthy^(26)^ |
| Brown | IR42 | 107 | 83* |  |  | Panlasigui and Thompson-2006-1 healthy^(26)^ |
| White | IR42 | 626 | 87 |  |  | Panlasigui and Thompson-2006-2-T2DM^(26)^ |
| Brown | IR42 | 406 | 56 |  |  | Panlasigui and Thompson-2006-2-T2DM^(26)^ |
| White | Basmati rice, 10 min | 94 | 50 |  |  | Ranawana-2009^(18)^ |
| Brown | Basmati rice, 25 min | 116 | 75 |  |  | Ranawana-2009^(18)^ |
| White | IR64 | 212 | 57 |  |  | Trinidad-2013^(30)^ |
| Brown | IR64 | 189 | 51 |  |  | Trinidad-2013^(30)^ |
| White | Sinandomeng | 280 | 75 |  |  | Trinidad-2013^(30)^ |
| Brown | Sinandomeng | 204 | 55 |  |  | Trinidad-2013^(30)^ |
